# Is the First of the Two Born Saved? A Rare and Dramatic Case of Double Placental Damage from SARS-CoV-2

**DOI:** 10.3390/v13060995

**Published:** 2021-05-26

**Authors:** Leonardo Resta, Antonella Vimercati, Sara Sablone, Andrea Marzullo, Gerardo Cazzato, Giuseppe Ingravallo, Giulia Mazzia, Francesca Arezzo, Anna Colagrande, Roberta Rossi

**Affiliations:** 1 Section of Pathology, Department of Emergency and Organ Transplantation (DETO), University of Bari “Aldo Moro”, 70124 Bari, Italy; leonardo.resta@uniba.it (L.R.); andrea.marzullo@uniba.it (A.M.); giuseppe.ingravallo@uniba.it (G.I.); g.mazzia@studenti.uniba.it (G.M.); anna.colagrande@gmail.com (A.C.); roberta.rossi@policlinico.ba.it (R.R.); 2 Section of Gynecology and Obstretics, Department of Biomedical Sciences and Human Oncology, University of Bari “Aldo Moro”, 70124 Bari, Italy; antonella.vimercati@uniba.it (A.V.); francescaarezzo@libero.it (F.A.); 3 Section of Legal Medicine, Department of Interdisciplinary Medicine, University of Bari, 70124 Bari, Italy; sarasabloneml@gmail.com

**Keywords:** SARS-CoV-2, placenta, COVID-19, fetus, autopsy

## Abstract

The current coronavirus pandemic has affected, in a short time, various and different areas of medicine. Among these, the obstetric field has certainly been touched in full, and the knowledge of the mechanisms potentially responsible for placental damage from SARS-CoV-2 occupy a certain importance. Here we present here a rare case of dichorionic twins born at 30 weeks and 4 days of amenorrhea, one of whom died in the first few hours of life after placental damages potentially related to SARS-CoV-2. We also propose a brief review of the current literature giving ample emphasis to similar cases described.

## 1. Introduction

The SARS-CoV-2 pandemic (Severe Acute Respiratory Syndrome Coronavirus-2) has had a global impact that has affected all different and distinct areas of medicine [1,2]. Among these, a place of great importance is occupied by the study and analysis of the effects of the virus on pregnant women. Although at the beginning of the pandemic there were few and anecdotal case reports or case series concerning these obstetric areas, an increasing number of scientific papers have tried to shed light on the mechanisms of etiopathogenesis and possible maternal–fetal transmission of the infection [3,4,5]. We present here a rare case of a COVID-positive woman, pregnant at 30 weeks + 4 days of amenorrhea, with a bi-chorial, bi-amniotic twin pregnancy, and with the birth of the first living and viable fetus and the birth of the second fetus with severe intra-partum distress and death after few minutes. We conducted morphological, immunophenotypic, electron microscopy and real-time polymerase chain reaction (RT-PCR) studies in order to confirm the presence of SARS-CoV-2 in placental tissues.

## 2. Materials and Methods

The patient was a 32-year-old woman with an early miscarriage previously suffering from mild hypothyroidism, 165 cm tall and 57 kg in weight. Approximately 2 weeks before labor, she contracted SARS-CoV-2, confirmed with a GeneXpert Dx Xpress SARS-CoV-2 RT-PCR assay (Cepheid). The analytical sensitivity and specificity are reported by the manufacturer as 100% (87/87 samples) and 100% (30/30 samples), with a limit of detection of 250 copies/mL or 0.0100 plaque-forming units per milliliter) [6], and she was experiencing modest symptoms, including cough, fatigue, headache, generalized malaise, and mild dyspnea without the need for mechanical ventilation. She was afebrile, heart rate (HR) = 100/min, respiratory rate (RR) = 14/min, blood pressure (BP) = 98 × 60 mmHg, oxygen saturation (SpO2) = 98% on room air, fetal heart rate (FHR) = 147/min and 113/min. She had been given antibiotic therapy for premature rupture of membranes (Prom) after a few days from maternal SARS-CoV-2 infection and prophylactic administration of corticosteroids for prematurity. Despite the administration of tocolytics, unstoppable labor was experienced at 30 weeks and 4 days of amenorrhea. Thus, preterm vaginal delivery occurred, and the first infant proved viable (weight 1295 g), while the second showed hypotonia and cyanosis and died after few minutes (weight 1340 g).

Placentas underwent routine clinical examination consisting of storage at 4 °C prior to fixation, fixation in 10% buffered formalin, photographs of the maternal and fetal surface, measurement, trimmed weight, sectioning, and examination of the cut surface. Sections submitted included 2 of membrane rolls, at least 2 of umbilical cord, 3 maternal surface biopsies, 2 full thickness sections, and representative sampling of any lesions present. Sections underwent routine processing, embedding, sectioning at 5 µm and staining with H&E. Histologic examination was performed by subspecialty perinatal pathologists who were aware of the COVID-19 status. Cases were reviewed by 2 pathologists to confirm the diagnoses. They were observed using an Olympus BX-51 optical microscope equipped with the Olympus DP80 image acquisition system. Anti-SARS-CoV-2 spike S1 glycoprotein monoclonal antibody, Thermo Fisher, Rabbit, was added, at pH 6, diluted 1:800, and the antigen was demonstrated by heat-induced citrate buffer epitope retrieval for enzymatic immunohistochemical (IHC) analysis. In addition, electronic microscopy analysis was made from villi samples of both the chorial discs. At the moment of delivery, samples were immediately fixed in 2.5% gluteraldehyde for 4 hours at 4 °C, and after overnight immersion in phosphate buffer, post-fixed with osmium tetroxide in PBS for 2 h at a temperature of 4 °C. The prepared samples were processed for inclusion in Araldite epoxy resin (M) CY212 (TAAB, Aldermason, UK). Semithin sections 0.5 µm thick were stained with toluidine blue for microscopic analysis. Ultrathin sections were mounted on nickel grilles with uranium acetate and lead citrate contrast. The semithin sections were observed with a Nikon photomicroscope equipped with a Nikon Coolpix DS-U1 digital camera (Nikon Instruments SpA, Calenzano, Italy). The ultrathin sections were observed with a Morgagni 268 electron transmission microscope (FEI Company, Naples, Italy).

## 3. Results

The first placental disc weighed 240 grams, measured 14 × 12 × 1.5 cm, had smooth and shiny membranes, and had a 31 cm umbilical cord, normospiralized, with paracentric insertion. The second disc weighed 340 g, measured 15 × 10 × 1.5 cm, had smooth and shiny membranes, and had a 15 cm, normospiralized funiculus with central insertion.

**Figure 1:**
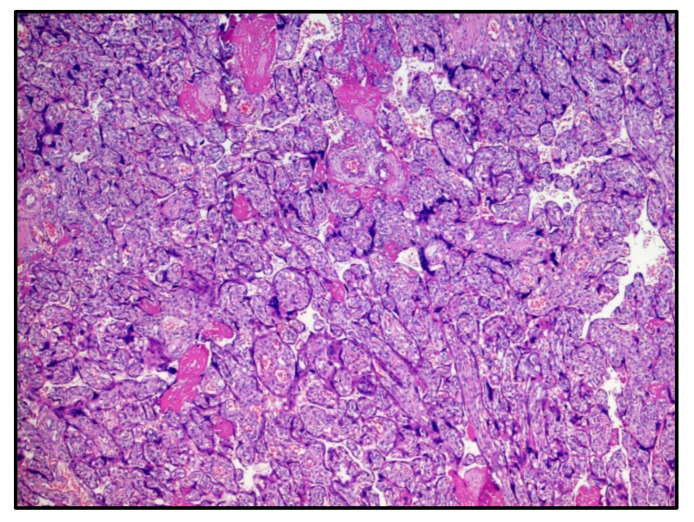
Histological features of the first chorionic disc: deposition of intervillous fibrin and chorionic villi corresponding to the gestational age (30 weeks). Hematoxylin-Eosin, 10×.

**Figure 2:**
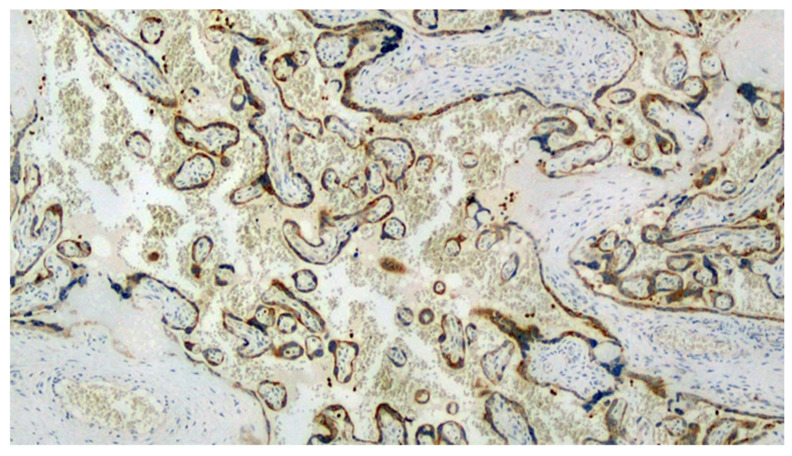
Immunostaining for anti-SARS-CoV-2 spike protein S1 positive at the level of the syncytiotrophoblast and perivillary histiocytes. (IHC, Original Magnification: 10×).

The first chorionic disc corresponded to the gestational age and presented very large areas of intervillous fibrinous deposition (Figure 1) with the presence of numerous perivillary histiocytes. Minor recent infarct foci were also described, while umbilical cord and amnio–chorionic membranes were completely normal. The immunohistochemical reaction for the SARS-CoV-2 protein S1 was strongly expressed both in the syncytiotrophoblast cells and in the perivillary histiocytes described in H&E (Figure 2).

**Figure 3:**
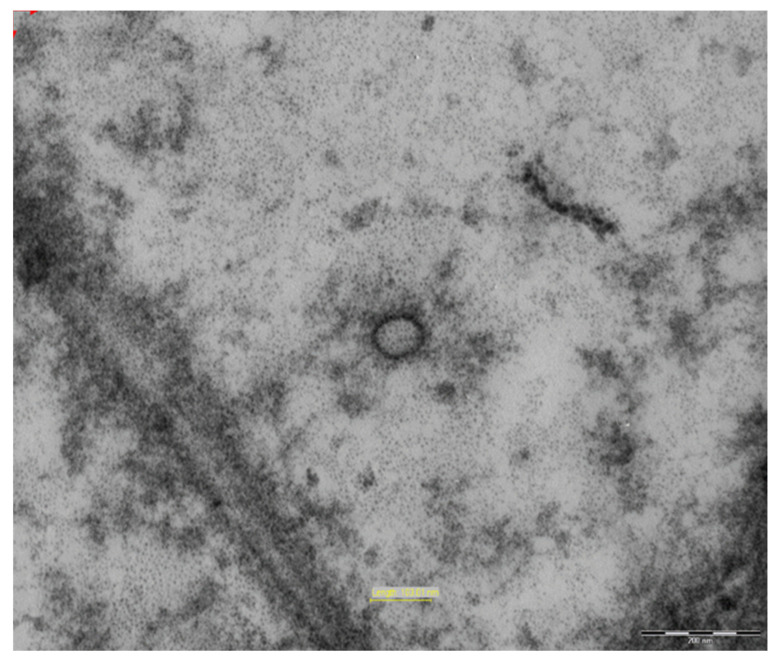
Circular formations with a 100–130 nm diameter were observed, with peripheral electron dense spicules, which are likely viral particles in the cytoplasm of the perivillary histiocytes. (Electron microscopy, 71,000×).

Electron microscopy showed signs of circular formations with a 100–130 nm diameter, with peripheral electron dense spicules, which are likely viral particles in the cytoplasm of the perivillary histiocytes (Figure 3).

**Figure 4:**
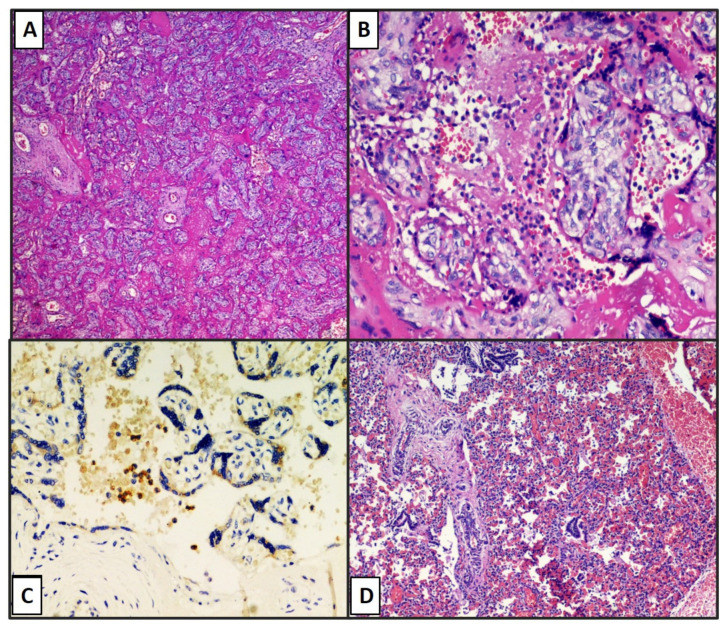
(**A**) Histology of the second chorionic disc with extensive and massive deposition of intervillous fibrin and presence of histiocytes available perivillary. (Hematoxylin–eosin, 4×). (**B**) Histological detail of the chorionic villi corresponding to the gestational age (30 weeks) with the presence of numerous histiocytes available perivillary (Hematoxylin–eosin, 20×). (**C**) Immunostaining for anti-SARS-CoV-2 S1 protein antibody strongly positive at the level of syncytiotrophoblast and perivillary histiocytes (brown staining, immunohistochemistry antiSARS-CoV-2 S1 spike protein, original magnification: 20×). (**D**) Histological examination of lung parenchyma of stillborn fetus with marked congestive phenomena and areas of peribronchial and interstitial hemorrhagic infiltration (hematoxylin–eosin, original magnification: 10×).

Additionally, in the second case, developmental characteristics corresponding to gestational age, large areas of intervillous fibrinous deposition (Figure 4A), and the presence of numerous perivillary histiocytes (Figure 4B) were described. Immunostaining for anti-SARS-CoV-2 S1 protein was strongly positive at the level of syncytiotrophoblast and perivillary histiocytes (Figure 4C).

Following the birth, the baby was subjected to a nasopharyngeal swab, which was positive, but which, when repeated after a few minutes, gave a negative result for SARS-CoV-2. Four days after death, a complete autopsy on the newborn was performed. On external examination, no malformations were detected. All-natural orifices were probed and appeared patent. Weight (1322 g) and anthropometric parameters were consistent with gestational age (30 w+ 4 d) in a twin pregnancy. External genitalia were normal and indicative of a male phenotype. By a skin and subcutaneous tissue Y-shaped incision, the thoracic and abdominal cavities were explored, noting that umbilical arteries normally extended on either side of the urinary bladder, along the inner abdominal wall, and the umbilical vein normally coursed towards the liver in the falciform ligament and patent. The domes of the diaphragm were inspected, and their positions were at the fourth rib interspaces bilaterally. After removing the chest plate, it was observed with a diaphanoscope, and four ossification nuclei were detected. Mild pleural, pericardial, and peritoneal effusions were noted. Lungs appeared hypo-expanded and crouched in their natural cavities. The heart only exhibited some subepicardial petechial spots on the anterior ventricular surfaces, and its opening in situ showed no congenital anomalies. After evisceration by the Rokitansky technique, all organs were carefully separated, dissected, and subjected to tissue sampling. At skull examination, normal tension of fontanels was appreciated. After their dissection, the brain was inspected in situ and then removed, revealing reduced tissue consistency due to decomposition and no focal lesions. Tissue samples were also taken from the brain. Histologically, all the organs removed, including the lung (Figure 4D), showed congestive and sometimes hemorrhagic phenomena. A focal epicardial lympho-monocytic infiltrate was also detected. A circumscribed area of coagulative necrosis was in the spleen beneath the capsule. For suspicion of the first positive swab, a histological lung sample was submitted to immunostaining for anti-SARS-CoV-2 S1 spike protein, which was totally negative. The data are summarized in Table 1.

## 4. Discussion

The new coronavirus-2 (COVID-19) pandemic has assumed a global importance that has affected various and distinct sectors of medicine [1,2]. Among these, the role of SARS-CoV-2 in placental pathology and the analysis of the risk of maternal–fetal transmission are of some importance [3,4,5]. In these months of the pandemic, different case reports and case series have tried to shed light on the placental histopathological alterations linked to the virus, and we ourselves have recently published a paper that reported the data of over 70 pregnancies of COVID-positive mothers [5]. The most significant finding is an increase in the rate of features of maternal vascular malperfusion (MVM), most prominently decidual arteriopathy including atherosis and fibrinoid necrosis and mural hypertrophy of membrane arterioles [5,7,8]. MVM, previously known as maternal vascular underperfusion, has been associated with oligohydramnios, fetal growth restriction, preterm birth, and stillbirth [9,10]. On the other hand, maternal–fetal transmission of the virus is a very rare event, with about 0.34% of cases described in the literature in COVID-positive mothers [7,11]. Our case is very important because it demonstrates once more how fetal discharge can be counted among the outcomes of SARS-CoV-2 infection. In this regard, recently, Poisson et al. [12] reported a rare case of fetal demise in a positive SARS-CoV-2 patient whose placental characteristics indicated extensive fetal vascular malperfusion with large infarct areas resulting in a rather substantial loss of the surface of the chorionic villi. Additionally, Baud et al. reported [13] a pregnant woman with symptomatic coronavirus disease who experienced a second-trimester miscarriage in association with documented placental SARS-CoV-2 infection. Richtmann et al. [14] reported their experience in Brazil of five SARS-CoV-2 positive women whose fetuses had died. Analysis of the placentas showed mainly moderate/severe chorionamniotitis, fetal thrombi, intervillous fibrin deposition, and intervillositis. More specifically, two cases had massive deposition of intervillous fibrin associated with mixed intervillitis and villitis, and intense neutrophil and lymphocyte T infiltration. Pulinx et al. [15] reported a more than rare case of vertical transmission of SARS-CoV-2 in a young 30-year-old woman from whose fetus had been collected various amniotic fluid samples at various times of pregnancy. Histological examination of placental tissues showed presence of extensive intervillous fibrin depositions and ischemic necrosis of the surrounding villi, together with aggregates of histiocytes and cytotoxic T lymphocytes in the intervillous space. In our case we observed severe placental alterations in a twin pregnancy. While in the first fetus these alterations did not compromise the viability, in the second, perhaps due to the prolongation of the expulsive phase and the ischemia of the contractions, the placental lesions (especially the intervillar fibrin and the activity of intervillar histiocytic cells) caused severe intra-partum suffering that irreversibly compromised the viability of the fetus. The relationship between COVID-related placental damage and neonatal death is possible, but this neonatal death can also result from other causes and factors.

## 5. Conclusions

Our case represents a rarity for two reasons: there was not the very rare maternal–fetal transmission of SARS-CoV-2 despite the placental parenchyma being strongly positive for immunostaining for the spike protein, but at the same time the damage to both placental discs did not allow the second fetus to be able to be adequately oxygenated in the intrapartum, leading to a disastrous outcome. We could sum it all up with “died of COVID without COVID”.

## Figures and Tables

**Table 1:** Features of twin pregnancy.

Characteristics	Fetus 1	Fetus 2
Condition	Born alive	Death after few minutes
Birth weight	1295 g	1340 g
APGAR score 1′–5′	9–10	1–1
Sex	Female	Male
First nasopharyngeal swab	positive	positive
Second nasopharyngeal swab	negative	negative
Placental Findings	Perivillous fibrin deposition	Perivillous fibrin deposition
	Perivillous histiocytes	Perivillous histiocytes
Anti-SARS-CoV-2 S1 spike protein	positive	positive

## References

[1] Jin Y., Yang H., Ji W., Wu W., Chen S., Zhang W., Duan G. (2020). Virology, Epidemiology, Pathogenesis, and Control of COVID-19. Viruses.

[2] Khan M., Adil S.F., Alkhathlan H.Z., Tahir M.N., Saif S., Khan M., Khan S.T. (2020). COVID-19: A Global Challenge with Old History, Epidemiology and Progress So Far. Molecules.

[3] Schwartz D.A. (2020). An analysis of 38 pregnant women with COVID-19, their newborn infants, and maternal-fetal transmission of SARS-CoV-2: Maternal coronavirus infections and pregnancy outcomes. Arch. Pathol. Lab. Med..

[4] Menter T., Mertz K.D., Jiang. S., Chen H., Monod C., Tzankov A., Waldvogel S., Schulzke S.M., Hösli I., Bruder E. (2020). Placental Pathology Findings during and after SARS-CoV-2 Infection: Features of Villitis and Malperfusion. Pathobiology.

[5] Resta L., Vimercati A., Cazzato G., Mazzia G., Cicinelli E., Colagrande A., Fanelli M., Scarcella S.V., Ceci O., Rossi R. (2021). SARS-CoV-2 and Placenta: New Insights and Perspectives. Viruses.

[6] Cepheid (2020). Xpert® Xpress SARS-CoV-2 Instructions for Use for Labs. https://www.fda.gov/media/136314/download.

[7] Shanes E.D., Mithal L.B., Otero S., Azad H.A., Miller E.S., Goldstein J.A. (2020). Placental Pathology in COVID-19. Am. J. Clin. Pathol..

[8] Zhou Y.Y., Ravishankar S., Luo G., Redline R.W. (2020). Predictors of High Grade and Other Clinically Significant Placental Findings by Indication for Submission in Singleton Placentas From Term Births. Pediatr. Dev. Pathol..

[9] Khong T.Y., Mooney E.E., Ariel I., Balmus N.C.M., Boyd T.K., Brundler M.-A., Derricott H., Evans M.J., Faye-Petersen O.M., Gillan J.E. (2016). Sampling and Definitions of Placental Lesions: Amsterdam Placental Workshop Group Consensus Statement. Arch. Pathol. Lab. Med..

[10] Redline R.W. (2005). Severe fetal placental vascular lesions in term infants with neurologic impairment. Am. J. Obstet. Gynecol..

[11] Hosier H., Farhadian S.F., Morotti R.A., Deshmukh U., Lu-Culligan A., Campbell K.H., Yasumoto Y., Vogels C.B., Casanovas-Massana A., Vijayakumar P. (2020). SARS-CoV-2 infection of the placenta. J. Clin. Investig..

[12] Poisson T.M., Pierone G. (2021). Placental pathology and fetal demise at 35 weeks of gestation in a woman with SARS-CoV-2 infection: A case report. Case Rep. Womens Health.

[13] Baud D., Greub G., Favre G., Gengler C., Jaton K., Dubruc E., Pomar L. (2020). Second-Trimester Miscarriage in a Pregnant Woman With SARS-CoV-2 Infection. JAMA.

[14] Richtmann R., Torloni M.R., Oyamada Otani A.R., Levi J.E., Crema Tobara M., de Almeida Silva C., Dias L., Miglioli-Galvão L., Martins Silva P., Macoto Kondo M. (2020). Fetal deaths in pregnancies with SARS-CoV-2 infection in Brazil: A case series. Case Rep. Womens Health.

[15] Pulinx B., Kieffer D., Michiels I., Petermans S., Strybol D., Delvaux S., Baldewijns M., Raymaekers M., Cartuyvels R., Maurissen W. (2020). Vertical transmission of SARS-CoV-2 infection and preterm birth. Eur. J. Clin. Microbiol. Infect. Dis..

